# Determination of post-pandemic impact on activities of daily living and instrumental activities of daily living factors affecting daily living activities of the elderly

**DOI:** 10.1590/1806-9282.20250683

**Published:** 2026-03-30

**Authors:** Rita Okwaameh Agyei, Nurcan Bilgic

**Affiliations:** 1 Private Hospital – Nicosia, Cyprus.; 2 World Peace University, Faculty of Health Sciences – Nicosia, Cyprus.

**Keywords:** Elderly, Activities of daily living, Pandemic

## Abstract

**OBJECTIVE:**

The aim of this study was to “determine post-pandemic impact on activities of daily living and instrumental activities of daily living factors affecting daily living activities of the elderly.” No difference was identified between the post-pandemic activities of daily living and instrumental activities of daily living levels of the elderly in the two countries.

**METHODS:**

The descriptive study was conducted in Ghana and Turkish Republic of Northern Cyprus between February 16 and July 1, 2022. A total of 390 elderly volunteers aged 65 and over living at home participated in this study. The participants were administered the sociodemographic data form, Katz Activities of Daily Living Scale, and Lawton and Brody Instrumental Activities of Daily Living Form. The Slovin formula was used in selecting the study’s sample. Slovin’s formula is used to calculate the sample size necessary to achieve a certain confidence interval when sampling a population.

**RESULTS:**

According to the activities of daily livings and instrumental activities of daily livings, they can continue their lives without being dependent on others, and the scale scores of women compared to men are statistically significant, and it can be said that women are less dependent than men.

**CONCLUSION:**

The study revealed that the pandemic had a negative impact on activities of daily livings and instrumental activities of daily livings. Participants’ physical activity since the pandemic had a negative impact on activities of daily livings and instrumental activities of daily livings. It may be recommended to develop appropriate policies so that elderly individuals can live without being dependent on others. In addition, it is recommended that a variety of scales measuring activities of daily living be adopted and adapted, which, when adapted, will provide more reliable results. Since this study was conducted as a cross-sectional study in only one region in two countries, it cannot be generalized to both countries.

## INTRODUCTION

Aging is a normal aspect of life. However, as people get older, their functional skills deteriorate, which results in poor health conditions and a reduction in their capacity to carry out their daily activities^1^.

In Ghana, the elderly population has increased sevenfold since the 1960 census, from 213,477 people in 1960 to 1,643,381 people in 2021^2^. Rural areas have a higher concentration of elderly people than urban areas. Only a few African nations, such as Ghana, have established policies aimed at protecting the health of the elderly^3^. Older individuals are free from paying premiums under the Ghanaian National Health Insurance Scheme (NHIS), which has been in existence since 2005^4^. According to the World Health Organization’s (WHO) NCD Country Profile for Ghana, communicable, maternal, perinatal, and nutritional diseases accounted for 53% of total mortality in the country across all ages, while NCDs accounted for 39% and injuries accounted for 8%. Men accounted for 49.8% of those who succumbed to NCDs, while women made up 36.4% of those who lost their lives to the diseases. In Ghana, cardiovascular disease, cancer, and respiratory disorders were the three leading causes of mortality from NCDs among those aged 60 and older and those of other ages. Furthermore, at least 19,883,528 doses of the COVID-19 vaccines have been given out in Ghana^5^.

An estimated 8% of the global population, 8.3% in Turkey, and 8.8% in the former Turkish Republic of Northern Cyprus (TRNC) are old. Aging is associated with an increase in acute and chronic health issues in the TRNC as well^6^. In the TRNC, 87.4% of the elderly were found to have at least one chronic condition, according to research. Hypertension affects 75.6% of the older population, whereas diabetes affects 41.5%. Heart-related disorders (24.5%), skeleton-related diseases (13.8%), vertigo (11%), and other chronic diseases (26%) were found in the study participants^7^. Those who are now old are also thought to be at risk for a wide range of mental health issues as a result of this historical process. The psychological issue was characterized by emotional isolation, sadness, and anxiety^6^. Elders in Northern Cyprus have a variety of alternatives for receiving healthcare, despite the lack of national health coverage^8^. The government provides public health facilities, including hospitals and health clinics. Those with social security insurance, which the government requires of everyone in the workforce, get a large portion of their medical expenses paid in public institutions. Elderly people over the age of 65 are eligible for free health care from the public sector in specific emergency circumstances and accidents. Additionally, at least 2,380,000 doses of COVID-19 vaccination had been given out by the TRNC as of this point^9^.

Functional capability or independence is a broad notion that encompasses activities of daily living (ADL). Subsequently, deficiencies in ADL influence everyday activities, job performance, and leisure activities. It is important to define the skill level, demonstrate the efficacy of rehabilitation, and determine a person’s capacity to do ADL^10^. Physical inactivity and social isolation are exacerbated by ADL^11^. Physical inactivity and social isolation increase frailty and aggravate chronic health conditions such as hypertension, cardiovascular and cerebrovascular disease, diabetes, depression, dementia, and coronavirus^12^.

The pandemic had a profound influence on elderly people, leading to physical inactivity and social isolation^13^. Public health interventions such as physical inactivity, social isolation, and lockdown were successful in preventing the spread of the virus. However, a decrease in social interaction outside the home may marginalize older persons^14^.

To fully understand the functional status of the elderly, ADL should be measured using different ADL instruments such as Katz and Lawton’s Instrumental Activities of Daily Living (IADL) scales^15^.

Numerous studies during the pandemic and post-pandemic recovery investigated the impact of a critical analysis of the pandemic and isolation implemented by different countries and its effect on the ADL of elderly people. Research has suggested that various factors influence ADL in the elderly using either the ADL index or the IADL scale^16^.

The importance of vaccination for the pandemic and other chronic diseases among older adults is frequently underestimated^17^.

The aim of this study is “determine post-pandemic impact on activities of daily living and instrumental activities of daily living factors affecting daily living activities of the elderly.”

### Hypothesis

H0: There is no difference between the post-pandemic ADL and IADL levels of the elderly in the two countries.

H1: There is a difference between the post-pandemic ADL and IADL levels of the elderly in the two countries.

### Research questions

1. What are the functional abilities of the aged?
2. What factors are affecting the Daily Living Activities of the elderly in their normal lives and during the pandemic?
3. Do the limitations to functional abilities as stated on the Katz ADL scale differ in its effect in Atibie, Ghana, and Degermenlik, TRNC?

## METHODS

### Study design and participants

This study employs a quantitative cross-sectional method for data collection. The study used a correlational research paradigm. It is possible to forecast future trends based on current information using the correlational research paradigm. The research employed a convenience sampling method. Convenience sampling uses the first suitable major data source without respect to objective conditions^18^. Convenience sampling does not utilize a predefined sample. Everyone is invited to participate. Slovin’s sample size formula was used in sample selection^19^.


$$\begin{array}{l} \text{n}=1+\frac{N}{1+Ne2}...\;\;...\;\;...3.1\\ \mathrm{thus:}\;N=\frac{N}{1+Ne2}...\;\;...\;\;...3.2 \end{array}$$

where n=no. of a selected sample,

N=total population,

e2=error margin/margin of error.

The universe of this research consisted of 146 people living in Kwahu Atibie Town, Ghana, and 1,400 people living in Değirmenlik Municipality, TRNC. A total of 390 elderly volunteers aged 65 and over living at home participated in this study, which was conducted using Slovin’s sample size of 110 people from Ghana and 280 people from the TRNC.

### Instruments

The data collection form includes sociodemographic characteristics (gender, age group, educational status, health status, and others), the Katz Index of Independence in Activities of Daily Living Skala, and the Lawton’s Instrumental Activities of Daily Living Scale.

The ADL Scale was developed by Katz et al.^20^, and the Cronbach’s alpha (α) value of the scale is 0.89^21^. In this study, the ADL Cronbach’s alpha coefficient obtained for the TRNC was 0.986 and for Ghana was 0.994. The results obtained in the TRNC and Ghana are higher than those obtained by Katz et al. Cronbach’s alpha values between 0.80 and 100 are considered highly reliable.

The IADL scale was developed by Lawton and Brody, and the Cronbach’s alpha value for the scale was found to range between 0.86 and 0.91^22^. In our study, the Cronbach’s alpha coefficient for the IADL for the TRNC was found to be 0.943 and 0.944 for Ghana. The Cronbach’s alpha obtained for the studies is consistent with the value obtained by Lawton and Brody^22^. The Cronbach’s alpha value for the pandemic activities for Ghana and the TRNC was found to be 0.81 and 0.78, respectively. Cronbach’s alpha value for both countries was greater than 0.70, indicating that the instruments used were reliable.

The WHO’s Study on Global Ageing and Adult Health (SAGE) project was designed to address this lack of information on aging in LMICs. One analysis from WHO-SAGE compared scores for functional difficulty on ADLs and IADLs across the 6 SAGE countries (Ghana, Mexico, South Africa, the Russian Federation, India, and China)^23^.

Data were collected between March 1, 2022, and April 30, 2022, from elderly people in the TRNC and Ghana. The questionnaire was in both English and Turkish languages. It was conducted face-to-face by nurses working in the health center in Ghana and by a Turkish-speaking nursing student in the TRNC.

### Ethics statement

Before the study commenced, ethics committee approval was acquired from the Ethics Committee, International Cyprus University (approval number: 020-4870, date: 10.05.2022), and necessary permissions were acquired from the mayor’s office. Volunteers were included in the study only if they gave informed consent to participate. Written permissions were obtained from the Değirmenlik Municipality and the Kwahu District Health Department. The study adheres to the principles of the Helsinki Declaration. All subjects provided informed consent prior to data collection.

### Statistical analysis

The study analyzed the data using the SPSS 24.0 package program. Descriptive data are numbers, percentages, mean, and standard deviation. Chi-square analysis was used in independent groups. p<0.05 was accepted as the significance limit.

## RESULTS

In our study, it was found that 70.7–71.8% of the participants in the TRNC and Ghana were between the ages 65 and 74, 51.4–51.8% were women, 48.6–46.4% were still actively working, 30.4–29.1% used any tobacco product, 55.4–57.3% had a chronic disease, and 89.6–90.9% had the influenza at least once in the last year.

In our study, it was found that 70.7–71.8% of the participants in the TRNC and Ghana were between the ages 65 and 74, 51.4–51.8% were women, 48.6–46.4% were still actively working, 30.4–29.1% used any tobacco product, 55.4–57.3% had a chronic disease, and 89.6–90.9% had the influenza at least once in the last year. The minimum age for both countries was 65–74 years, and the average age was also 65–74 years. The highest age for the respondents in both countries was 85 years or older. The median value for TRNC was 65–74 years and that of Ghana was 65–74 years.

In both countries, the probability values which were less than (p<0.005) were obtained for the questions “which of these vaccination(s) have you had within the last 5 years,” “reason for vaccination,” “reason for being unvaccinated,” “factors that influence your decision to be vaccinated.” This shows that the vaccination status of respondents in both countries was statistically significant (Table 1).

**Table 1:** Sociodemographic and vaccination-related characteristics of the elderly (n=390).

Responses	TRNC			Ghana		
	n (280)		%	n (110)		%
Age group						
65–74 years	198		70.7	79		71.8
75–84 years	63		22.5	22		20.0
85+	19		6.8	9		8.18
Gender						
Males	136		48.6	53		49.2
Females	144		51.4	57		51.8
Educational status						
Informal education	61		21.8	23		20.9
Primary	129		46.1	51		46.4
Secondary	73		26.1	29		26.4
Graduate	17		6.2	7		6.4
Marital status						
Single	14		5.0	5		4.5
Married	178		63.6	70		63.6
Divorced	49		17.5	20		18.2
Widowed	39		13.9	15		13.6
Profession status						
Active	136		48.6	51		46.4
Retired	144		51.4	59		52.6
Living region						
District	12		4.2	4		3.6
Village	268		95.7	106		96.4
Which of these vaccination(s) have you had within the last 12 months?						
Hepatitis B	9	3.2	p=0.763	4	3.6	p=0.763
Tetanus	6	2.1		1	0.9	
COVID-19	262	93.6		104	94.5	
MMR	3	1.1		1	0.9	
Which of these vaccination(s) have you had within the last 5 years?						
Hepatitis B	9	3.2	p=0.000	4	3.6	p=0.000
Tetanus	6	2.1		1	0.9	
MMR	3	1.1		1	0.9	
Others	262	93.6		104	94.5	
Reason for being unvaccinated						
Believing that it is not necessary	156	55.71	p=0.000	53	48.18	p=0.000
Having inadequate information	98	35		23	20.91	
Ineffective previous vaccination	5	1.79		11	10	
Cost concern	21	7.5		23	21.90	

p<0.005. TRNC: Turkish Republic of Northern Cyprus.

The ADL subscales of the participants in both countries were found to be statistically significant, and it was found that elderly individuals were able to bathe, dress, go to the toilet, transfer, control urine, and feed themselves (p<0.005) (Table 2).

**Table 2:** Status of participants’ activities of daily living and instrumental activities of daily living scale scores (n=390).

ADLs responses		TRNC (n=280)			Ghana (n=110)		
		n	%	p-value	n	%	p-value
	Dependent	21	7.5		10	9.1	
Dressing	Independent	263	93.9	0.000	100	90.9	0.000
	Dependent	17	6.1		10	9.1	
Toileting	Independent	258	92.1	0.000	101	91.8	0.000
	Dependent	22	7.9		9	8.2	
Transferring	Independent	258	92.1	0.000	102	92.7	0.000
	Dependent	22	7.9		8	7.3	
Continence	Independent	261	93.2	0.000	101	91.8	0.000
	Dependent	19	6.8		9	8.2	
Feeding	Independent	263	93.9	0.000	104	94.5	0.000
	Dependent	17	6.1		6	5.5	
**IADL responses**							
Ability to use telephone	Independent	213	76.1	0.000	84	76.4	0.000
	Dependent	67	23.9		26	23.6	
Shopping	Independent	210	75	0.000	83	74.5	0.000
	Dependent	70	25		27	24.5	
Food preparation	Independent	223	79.4	0.000	62	56.4	0.000
	Dependent	57	20.4		48	43.6	
Housekeeping	Independent	208	74.3	0.000	82	74.5	0.000
	Dependent	72	25.7		18	25.5	
Laundry	Independent	204	72.9	0.000	81	73.4	0.000
	Dependent	76	29.1		29	26.4	
Mode of transport	Independent	172	61.4	0.000	67	60.9	0.000
	Dependent	108	38.6		43	39.1	
Responsibility for own medications	Independent	209	74.6	0.000	83	74.5	0.000
	Dependent	71	25.4		27	24.5	
Finance handling	Independent	201	71.8	0.000	79	71.8	0.000
	Dependent	79	28.2		31	28.2	

p<0.005. TRNC: Turkish Republic of Northern Cyprus; ADLs: activities of daily livings; IADL: instrumental activities of daily living.

Table 3 shows that there has not been much change in the physical activities of the participants in the TRNC and Ghana since the pandemic; they generally preferred walking to do physical activity during the pandemic. While the participants in the TRNC frequently met with their neighbors, they did physical activity at home in Ghana, and this was found to be statistically significant (p<0.005).

**Table 3:** Pandemic activities of the elderly who participated in the study (n=390).

Responses			TRNC			Ghana		
			n (280)	%	p-value	n (110)	%	p-value
Has there been any changes in physical activity since the pandemic?								
More			52	18.6	0.000	20	18.2	0.000
About the same			213	76.1		88	80.0	
Less			15	5.3		2	1.8	
What is the most common type of physical activity you perform?								
Walking			225	80.4	0.000	85	77.3	0.000
Jogging/running			26	9.3		10	9.1	
Other (biking/cycling/weight training/online videos/classes/yoga/home workout/hiking)			29	10.3		15	13.6	
What is your most common physical activity location?								
In the house			51	18.2	0.000	61	55.5	0.000
Neighborhood			151	53.9		30	27.3	
Recreational sites, trails, parks			72	25.7		13	11.8	
Others			6	2.1		6	5.5	
Did you have access to this physical activity location during the pandemic?								
Yes			197	70.4	0.000	83	75.5	0.000
No			83	29.6		27	24.5	
**Regression result**	**ADL (TRNC)**		**ADL (Ghana)**		**IADL (TRNC)**		**IADL (Ghana)**	
	**SE**	**p-value**	**SE**	**p-value**	**SE**	**p-value**	**SE**	**p-value**
(Constant)	1.343 (0.444)	0.003	1.181 (0.925)	0.204	-0.162 (0.436)	0.710	-0.201 (0.882)	0.821
Age group	0.326 (0.034)	0.000	0.356 (0.059)	0.000	0.239 (0.033)	0.000	0.241 (0.056)	0.000
Gender	0.035 (0.035)	0.312	0.042 (0.058)	0.476	0.010 (0.034)	0.774	0.009 (0.055)	0.874
Educational status	-0.064 (0.021)	0.003	-0.065 (0.035)	0.069	0.041 (0.021)	0.048	0.036 (0.034)	0.294
Marital status	-0.010 (0.024)	0.697	-0.028 (0.042)	0.510	0.047 (0.024)	0.052	0.041 (0.040)	0.311
Professional status	-0.044 (0.038)	0.245	-0.029 (0.063)	0.649	0.103 (0.038)	0.006	0.119 (0.061)	0.053
Living region	-0.064 (0.140)	0.645	-0.032 (0.292)	0.913	0.234 (0.137)	0.089	0.260 (0.279)	0.354
Number of observations	280		110		280		110	
R-square	0.355		0.364		0.354		0.330	
Adjusted R-square	0.332		0.305		0.332		0.267	
F-test	15.993 (0.000)		6.167 (0.000)		15.860 (0.000)		5.260 (0.000)	

p<0.005, F-test, R-square, adjusted R-square. ADL: activities of daily living; TRNC: Turkish Republic of Northern Cyprus; IADL: instrumental activities of daily living; SE: standard error.

In the TRNC, age was found to have a positive effect on ADL. The age group of the respondents was seen to have a statistically significant effect on ADL. In Ghana, age has a positive effect on ADL. The age group of the respondents has a statistically significant effect on ADL. The age groups of 65–74 years, 75–84 years, and 85+ years were found to have a statistically significant effect on ADL and IADL in both countries. Gender has a positive effect on ADL in both countries (TRNC and Ghana). There is no statistically significant effect of gender on ADL in either country.

The educational status of the respondents has a negative effect on ADL in both countries. However, in the TRNC, the educational status of the respondents was found to have a statistically significant impact on ADL, but in Ghana, the educational status of the respondents was not found to have a statistically significant effect on ADL. The marital status of the respondents has a negative effect on ADL in both countries. The marital status of the respondents did not have a statistically significant impact on ADL in both countries.

The R-square explains the variation in the ADL and IADL explained by the sociodemographic of the respondents. In the TRNC, the sociodemographic of the respondents explained ADL and IADL by 36 and 35%, and in Ghana, the sociodemographic of the respondents explained ADL and IADL by 36 and 33%. The F-tests measure the joint impact of the sociodemographic characteristics of the respondents on ADL and IADL. The results indicated that in both countries, the sociodemographic characteristics of the respondents had a joint impact on the ADL and IADL. The Durbin–Watson measures the autocorrelation between the sociodemographics of the respondents and the ADL and IADL. The results show that there is a positive autocorrelation between the sociodemographics of the respondents and the ADL and IADL.

## DISCUSSION

According to Pasanen et al., in the EU-27, older women (65 years of age or older) made up 40% of the population who lived alone in 2018, compared to older men (22%). Less than one-third (31%) of adults of working age lived in under-occupied housing in the EU-27 in 2018, compared to close to half (47%) of older adults (65 years or older) (aged 18–64 years). In 2019, the EU-27’s older women (75 years of age or older) were more likely than older men to have significant financial hardships, with the rate of severe material deprivation for older men being 3.3% and for older women being 6.0%^24^.

There is still room for development, though. Improving knowledge of and treatment rates for dyslipidemia and diabetes will help decrease the 38% gap in heart disease mortality between older men and women^25^.

Most vaccines are made specifically for children and young adults, whose immune systems differ from those of older people, in whom physiological immune suppression coexists with a personal history of diseases and vaccinations^26^. According to WHO, seasonal influenza may cause between 290,000 and 650,000 fatalities annually due to respiratory illnesses^27^. According to WHO ranking of influenza and pneumonia, Ghana was ranked as the 26th country with higher recording of influenza and pneumonia, while Cyprus was ranked 182nd. Age affects the efficacy of the influenza vaccine, which protects against the disease between 70 and 90% in children and adults but drops to 30–50% in people over 65^28^. According to the WHO’s 2022 data, the rate of Covid-19 vaccination per 100 people in Ghana is 45.0 and 75.0% for the TRNC. The proportion of people who have received at least one dose of the COVID-19 vaccine is 45% in Ghana and 75% in the TRNC (having received a minimum of one dose of a COVID-19 vaccine)^27^. Similarly to how responses to hepatitis B and pneumococcal polysaccharide vaccinations are affected by aging, older individuals experience shorter-lasting antibody responses^29^. According to Young-Xu et al.^30^, at least one dose of the covid-19 vaccine has been administered to 68.4% of the world’s population. There were 12.86 billion doses that were administered across the world, and there are currently 2.69 million doses administered each day. In countries with a low per capita gross domestic product (GDP), 23.3% of the population has received at least one dose. The reasons why they were vaccinated are protection against diseases, which is officially recommended for people aged 65 years and above, and reducing the risk of chronic diseases. At least 19,883,528 doses of the covid-19 vaccines have been given out in Ghana^27^. Additionally, at least 2,380,000 doses of covid-19 vaccination had been given out by the TRNC as of this point. This study did not investigate vaccination information by age or health status. Because the data is self-reported, vaccination rates are believed to be high.

Table 2 shows that the participants in both countries had statistically significant IADL subscales and that elderly individuals were able to use the telephone, shop, prepare meals, do housework, do laundry, take their own medications, use transportation, and manage nutrition and financial affairs on their own. The IADL scale measures eight different functional domains. It is therefore crucial to consider the adaptation of country-specific scales when doing such analysis and was applied by Isik et al. in adapting Lawton’s IADL Turkish^31^.

Across all countries, functional difficulty scores increased with age and were higher for women. Age-specific functional difficulty scores were lowest in China, indicating lower functional difficulty among older adults, and the highest in India, with South Africa and Ghana having the next highest scores. Approximately 90% of people aged 60 years and older in Ghana had functional difficulties across the ADL and IADL items assessing cognition, mobility, self-care, getting along with people, engagement in household responsibilities, and participation in society (Biritwum). It is thought that our study is not similar to the literature because the data is based on declaration, and elderly individuals may have problems remembering during the pandemic period.

Even though the study attempted to compare the daily activities of both the TRNC and Ghana using the IADL scale, it is crucial to state that both countries have significantly diverse cultures that will uniquely influence their physical output and performance in some of their daily activities. It is therefore crucial to consider the adaptation of country-specific scales when doing such analysis, and was applied by Isik et al. in adapting Lawton’s IADL to Turkish^31^. And also the WHO’s SAGE project was designed to address this lack of information on aging in LMICs^23^. One analysis from WHO-SAGE compared scores for functional difficulty on ADLs and IADLs across the six SAGE countries (Ghana, Mexico, South Africa, the Russian Federation, India, and China)^23^.

Patel et al. examined functional disability among older adults in India^32^. They found that age and sex have a positive effect on IADLs and educational status, marital status, and professional status have a negative effect on IADLs. Both findings are backed by the study’s results. Some research has examined IADL and their implications for older adults’ oral health in Brazil. They found that age, marital status, professional status, and educational status have a positive effect on ADLs. They also found that sex has a negative effect on ADLs. The findings that age has a positive effect on ADLs is the same as the study’s results^33^. Jang, Ko, and Han found that age, marital status, and education status have a positive effect on ADLs and support the study’s results that age has a positive effect on ADLs, but the rest of the findings are not congruent with the study’s results^34^.

Pandemic activities had a positive relationship with the age group, number of children, communication with children, and professional status of the respondents. There is a negative relationship between pandemic, gender, educational status, marital status, accommodation type, and the living region of the respondents. Portela et al. found that age and gender have a positive and significant effect on ADLs^35^.

The relationship that exists between the pandemic and the age and gender of the elderly is not the only subject that has been researched. Many other studies have also documented the negative impact of the pandemic and its resultant lockdown on the physical ability of the elderly, which is in line with the results from the current study. According to Nejadsadeghi et al., the inactivity and isolation experienced by the elderly resulted in a medical condition known as sarcopenia^36^. The study further explained that the physical immobility among older people is what is responsible for the worsening age-related muscle function and strength.

Again, the negative impact of inactivity on the health of the elderly has also been supported by Pashmdarfard et al., who concluded that isolation and inactivity are detrimental to the health of the elderly due to the absence of muscle exercise. Sharing some revealing statistics on the consequences of immobility of adults and its health-related consequences, a situation predominantly experienced during pandemic^37^. Nejadsadeghi et al. posited that not only could 5–10 days of limb inactivity and bed rest result in muscle atrophy, but also increased inactivity among the elderly simultaneously increases their mortality^36^.

The relationship that exists between the pandemic and the age and gender of the elderly is not the only subject that has been researched. Many other studies have also documented the negative impact of the pandemic and its resultant lockdown on the physical ability of the elderly, which is in line with the results from the current study.

Again, the negative impact of inactivity on the health of the elderly has also been supported by Pashmdarfard et al., who concluded that isolation and inactivity are detrimental to the health of the elderly due to the absence of muscle exercise^37^.

## CONCLUSION

The studies revealed that the pandemic had positive and negative effects on ADLs and IADLs. The physical activities of the respondents since the pandemic had a negative effect on IADL.

The study provides an overview of ADL and IADL competencies, but does not fully explain the factors that influence these competencies. However, this would be an interesting topic for future research. Therefore, appropriate and relevant knowledge, skills, and attitudes should be provided to older adults through peer and community education to enable them to develop these competencies.

### Limitations

This study’s limitations include the fact that it was conducted in a single region in both countries and that the forms used for data collection were not validated and reliable. The study’s reliance on self-reporting and a cross-sectional descriptive design makes it difficult to generalize to the population.

## Data Availability

The datasets generated and/or analyzed during the current study are available from the corresponding author upon reasonable request.
